# Effects of BIO on proliferation and chondrogenic differentiation of mouse marrow-derived mesenchymal stem cells

**Published:** 2013

**Authors:** Mohamadreza Baghaban Eslaminejad, Nasrin Fallah

**Affiliations:** 1 *Department of Stem Cells and Developmental Biology at Cell Science Research Center, Royan Institute for Stem Cell Biology and Technology, Avicenna Research Institute, Tehran, Iran; *; 2 *Department of Developmental Biology, University of Science and Culture, Tehran, Iran.*

**Keywords:** BIO, Cartilage differentiation, Mesenchymal stem cells, Mouse, Proliferation

## Abstract

*In vitro* expansion of mesenchymal stem cell (MSCs) into large number is necessary for their application in cell-based treatment of articular cartilage defects. On the other hand, some studies have indicated that BIO (6-Bromoindirubin-3-Oxime) possesses mitogenic effects on cell culture. The objective of the present study was to examine the effect of BIO on in vitro expansion and chondrogenic differentiation of mouse marrow-derived MSCs. The culture was established using bone marrow tissue obtained from 10 NMRI mice. MSC nature of the isolated cells was verified according to the minimal criteria proposed for MSC. Passaged-3 cells were seeded in 24-well culture plates and treated by 0.05, 0.01, 0.1, 1.0 and 1.5 µM BIO for seven days. The culture without BIO was taken as the control. At the end of cultivation period, the cultures were examined for viable cell number which was then used to calculate population doubling time (PDT). The BIO with higher proliferation-promoting effect was investigated for its chondrogenic effect on MSC culture. There was significantly more viable cells at the cultures treated by 0.1 µM BIO. At this culture the cells tended to double their population in rapid rate (each 43.07 hr) than the cells treated with the other BIO concentrations (*p* < 0.05). Interestingly treatment of MSC chondrogenic culture with 0.1 µM BIO led to the up-regulation of cartilage specific genes including aggrecan, collagen II and Sox9. In conclusion BIO at 0.1 µM could enhance mouse MSC in vitro proliferation as well as their chondrogenic differentiation. These findings would be of great importance for the field of regenerative medicine.

## Introduction

Mesenchymal stem cells (MSCs) are defined as adult stem cells capable of undergoing renewal division for a relatively long time and the potential of differentiation into skeletal cell lineages under an appropriate culture condition.^1^^,^^2^ The first definitive evidence of MSC existence has been provided by Friedenstein* et al*.^3^ These investigators plated marrow cells in plastic dishes and removed non-adherent cells four hr after culture initiation. Friedenstein *et al*. have reported that a small fraction of the adherent cells were heterogeneous producing small cell aggregates with spindly-shaped morphology. The most important feature of the cells was their capacity to produce small deposits of bone and cartilage-like tissue. These authors have referred to the cells as colony forming unit-fibroblast (CFU-F).^3^^,^^4^ The following investigations have confirmed and developed preliminary observations of Friedenstein *et al*. Nowadays, CFU-F cells introduced by Friedenstein *et al*. are often called as mesenchymal stem cells.^5^^-^^7^ MSCs are considered as an appropriate cellular material for promoting regeneration in injured tissues in particular articular cartilage tissue. The repair of this tissue is impaired since it lacks blood vessel which is necessary for repair process.^8,^^9^

Studies have indicated that MSCs occur in a very low frequency (10-15 cells per 10^6^ marrow nucleated cells) in bone marrow samples.^1^ For this reason, *ex vivo* expansion of the cells is an inevitable task prior to any either experimental work or clinical setup. The routine culture technique for expanding MSCs is to use a medium containing 10-15% fetal bovine serum (FBS).^10^^,^^11^ Under these conditions cells undergo a reasonable proliferation leading to a cell yield that is proportional to the volume of marrow samples used to initiate the culture. On the other hand, at cell-therapy strategy, a huge number of stem cells are required.^12^^,^^13^ To achieve this number, it will be necessary to obtain a large volume of marrow aspirates as a starting material of culture initiation.^12^^,^^13^ Since the obtainable volume of marrow is limited, finding a culture condition favoring the MSC proliferation could be of great importance.

One strategy to enhance the *ex vivo* expansion of MSC is to manipulate the molecular pathway involved in cell proliferation. Wingless-type MMTV (mouse mammary tumor virus) integration site family of the protein (Wnt) signaling pathway is among those pathways governing cell proliferation. The canonical Wnt pathway is initiated by binding of Wnts to frizzled receptors and their co-receptors are called as low-density lipoprotein receptor-related proteins 5 and 6 (LRP5/6) and followed by activation of Dishevelled family proteins (DsH) which is a key component of a membrane-associated Wnt receptor complex. Activation of DsH inhibits a second complex of cytoplasmic proteins that include axin, GSK-3 (glycogen synthase kinase-3), and the protein APC (adenomatous polyposis coli). The inhibition of this complex leads to the entrance of beta catenin into the nucleus and activating Wnt-responsive genes. At the absence of Wnt proteins, beta catenin is phosphorylated and rapidly destructed by ubiquitin-proteaosome.^14^^-^^16^

Some works has indicated that BIO (6-bromoindirubin-3-oxim) can play as GSK-3β inhibitor mimicking the action of Wnt secretive molecules.^17^ BIO is a derivative of indirubin that is obtained from a trypan purple. It adheres on a groove between ATP and GSK-3β and inhibits GSK-3β resulting in activation of Wnt signaling pathway. The effect of this reagent has so far been investigated on various cell culture including hypocampal cells,^18^ epithelial cells from kidney proximal tubule,^19^ and human and murine embryonic stem cell.^20^ In previous investigation we studied the effect of BIO on MSC derived from rat bone marrow and indicated its proliferation promoting effects.^21^ Since MSCs from different species may behave differently, in the present study, we investigated the effect of BIO on MSC from mouse bone marrow. Furthermore, in this study, chondrogenic effect of BIO was examined.

## Materials and Methods

**Bone marrow cell culture. **Ten male NMRI mouse were included in this study. The use of animal was approved by ethic committee of Royan Institute, Tehran, Iran. The animals were sacrificed by cervical dislocation and their tibia and femur were collected. Under sterile condition, bone marrow from the long bones was flushed out using an insulin needle inserted into the clipped end of the long bones. The samples was mixed with 5 mL DMEM (Dulbecco’s Modified Eagle Medium, Gibco, Paisley, UK) containing 15% FBS (Gibco, Paisley, UK) and 100 IU penicillin (Gibco, Paisley, UK) and 100 µg mL^-1^ streptomycin (Gibco, Paisley, UK). The solution was centrifuged for 3 minute at 400 *g*. Supernatant was removed and the pellet was suspended in medium and plated at 10^6^ cells per mL in 75 cm^2^ culture flasks. The cultures were incubated in an atmosphere of 5% CO_2_ and 37 ˚C temperature. Three days after culture initiation, the medium was replaced with a fresh medium. The medium substitution was performed twice weekly till confluency reached. At this time the cultures were trypsinized and subcultured in 1:3 ratio. Passaged-3 cells were used at the following experiments.

**Flow cytometry of cell surface markers. **To examine whether some known MSC markers was present/absent on the isolated cells, flow cytometric analysis was performed using PE (phycoerythrin)-conjugated CD73, CD44, CD34, CD11b and CD31. In brief, 10^6^ passaged-3 cells were placed in 5 mL tubes, 5 µL of either phycoerythrin (PE) or fluorecein isothiocyanate (FITC)-conjugated antibody and 5 µL of blocking buffer were then added. The cells were incubated at 4 ˚C for 20-25 min at a dark place, followed by washing with 1 mL washing buffer (PBS supplemented with 1% FBS) and centrifugation at 400 *g*. The cell pellet was then suspended in 300-500 µL washing buffer and analyzed by flow cytometry (FACSCalibur cytometer equipped with 488 nm argon lasers (Becton Dickinson, Franklin Lakes, NJ, USA). In this study, Immunoglobulin G_2_ (IGG_2_) and Immunoglobulin G_1_ (IGG_1_) were used as the isotope control. WinMDI software (Version 2.8, Microsoft Corporation, Redmond, WA, USA) was used to analyze the flow cytometric results. 

**Multilineage differentiation. **For osteogenic differentiation, confluent culture of passaged-3 cells were provided with DMEM supplemented with 50 mg mL^-1^ ascorbic 2-phosphate (Sigma, St. Louis, MO, USA), 10 nM dexamethazone (Sigma, St. Louis, MO, USA) and 10 mM β-glycerol phosphate (Sigma, St. Louis, MO, USA) for three weeks. At the end of this period, the cultures were stained by alizarin red for mineralized matrix. RT-PCR was also performed to detect osteo-specific gene expression.

For adipogenic differentiation, likewise, the confluent cultures were treated by induction medium consisting of DMEM supplemented with 50 μg mL^-1^ ascorbic acid 3-phosphate, 100 nM dexamethazone and 50 μg mL^-1^ indomethacin. After three weeks, the cultures stained by Oil red for lipid droplet. Moreover RT-PCR was done to verify whether or not adipose-specific gene was expressed at the differentiated cultures. 

For chondrogenic differentiation, 2.5 × 10^5^ passaged-3 cells were pelleted under 400 *g* for 5 min and provided with DMEM supplemented with 10 ng mL^-1^ transforming growth factor β3 (TGF-β3 Sigma, St. Louis, MO, USA), 10 ng mL^-1^ bone morphogenetic protein-6 (BMP6, Sigma, St. Louis, MO, USA), 50 mg mL^-1^ insulin transferin selenium + premix (Sigma, St. Louis, MO, USA), 1.25 mg bovine serum albumin (Sigma, St. Louis, MO, USA) and 1% FBS for three weeks. At the end of this period, the pellets were histologically prepared, embedded in paraffin wax, cut into 5 μm thick sections and stained by toluidine blue. Some pellets were used to extract mRNA in order to further examine cartilage-specific gene expression by RT-PCR.

**RT-PCR (Reverse-transcription-polymerase chain reaction) analysis. **Total RNA was isolated from the differentiated cells of the osteogenic, adipogenic and chondrogenic cultures using the RNX^TM^-Plus (RN7713C; CinnaGen Inc., Tehran, Iran). In order to eliminate residual DNA, the RNA sample was treated with 1 U µL^-1^ of RNase-free DNaseI (EN0521; Fermentas, Opelstrasse, Germany) per 1 µg of RNA in the presence of 40 U µL^-1^ of ribonuclease inhibitor (E00311; Fermentasm, St. Leon-Rot, Germany) and 10× reaction buffer with MgCl_2_ for 30 min at 37 ˚C. DNaseI was inactivated by adding 1-2 µL of 25 mM EDTA and incubation at 65 ˚C for 10 min. Standard RT reactions were performed with 2 μg total RNA using random hexameras, a primer and a RevertAid First Strand cDNA Synthesis Kit (Fermentas, St. Leon-Roth, Germany) according to the manufacturer’s instructions. For every reaction set, one RNA sample was prepared without RevertAid MMuLV reverse transcriptase (RT-reaction) in order to provide a negative control of the subsequent PCR. To minimize variation in the RT reaction, all RNA samples from a single experimental setup were simultaneously reverse-transcribed. Reaction mixtures for PCR included 2 µL cDNA, 10xPCR buffer(AMSTM; CinnaGen Co., Tehran, Iran), 200 mM dNTPs,1.5-2 mM MgCl_2_ (CinnaGen Inc., Tehran, Iran), 0.5 mM of each antisense and sense primer (Table 1) and distilled water up to reach to the total volume. The RT-PCR reaction was performed in 25 µL.

**Culture treatment by BIO. **After ensuring that the isolated cells meet the minimal criteria of MSCs including having the multilineage differentiation potential and ex-pressing some certain surface markers, the main experiment was begun. First passaged-3 cells were plated at 10^4^ cells per wells in 24-well culture plate. Two days after culture initiation, the medium were removed and a fresh medium containing different concentrations of BIO including 0.01, 0.05, 0.1, 1.0 and 1.5 µM was added. These concentrations were selected according to the previous investigations.^17^^,^^18^ The culture without BIO was taken as the control group. A week after treatment, when at least one group achieved confluency, the cultures were terminated and examined for viable cell number using MTT assay.

**MTT (3-(4,5-dimethylthiazol-2-yl)-2,5-diphenyl tetra-zolium bromide) assay. **MTT (Sigma, St. Louis, MO, USA) mitochondrial reaction is an assay that is based on the ability of live cells to reduce a tetrazolium-based compound, MTT, to a purplish formazan product. In brief, the cultures were washed with PBS, added with the solution composed of 5:1 ratio of media and MTT solution (5 mg mL^-1^ in PBS), and incubated for 2 hr at 37 ˚C. Medium and MTT solution was removed and 0.5 ml extraction solution (Dimethyl-sulphoxide: DMSO) was added to solve the foramazone precipitate. The absorbance of the supernatant was read with a microplate reader (BioTek ELx 800, Bedfordshire, UK) at 540 nm. Cell number was determined through a standard curve that was established by using a known number of cells counted by a coulter counter. 

**Population Doubling Time (PDT). **Population doubling time is defined as the time during which the number of cells doubles in culture. To exactly determine BIO effect on MSC *in vitro* propagation, this valuable index was calculated for the cultures treated with BIO. Population doubling time was determine by the equation “PDT = Culture duration/ Population doubling number (PDN)”. To calculate PDN, the equation “PDN = (logN/N_0_×3.31)” was used.^22^ In this equation N and N_0_ are numbers of cells at the end and beginning of the culture period, respectively. In this study the culture period for determining PDT was seven days.

**Chondrogenic culture using BIO. **To evaluate whether the best mitogenic concentration of BIO (determined at above experiment) was effective at murine MSC chondrogenic differentiation, a cartilage induction culture was established by passaged-3 mouse MSCs and was added to the culture medium. The culture supplemented with an ordinary chondrogenic medium (see section “multilineage differentiation”) was taken as the control. All cultures were maintained for a 3-week period. At the end of the period, the cultures were quantified for up-regulation/down-regulation of cartilage-specific genes including aggrecan, collagen II and sox 9. 

**Quantitative real-time RT-PCR. **To quantify relative gene expression levels in chondrogenic cultures with and without BIO treatment, total RNA was extracted from the cultures using Trizol (Invitrogen, Paisley, UK). cDNA was synthesized from total RNA using a RevertAidTM First Strand cDNA Synthesis Kit (Fermentas, St. Leon-Roth, Germany) according to the manufacturer’s instructions. Aggrecan, collagen II and Sox9 mRNA levels as a chondrogenic differentiation marker genes, were measured by real-time RT-PCR (StepOne™ real-time PCR Applied Bio-systems, Foster City, CA, USA). The 20-μL reaction contained 2 μL cDNA from each sample mixed with 10 μL SYBR^®^ Green PCR Mastermix (Invitrogen, Paisley, UK), 2 μL primers and 6 μL RNase/DNase-free water. The PCR conditions were: incubation at 95 ˚C for 2 min followed by 45 cycles at 95 ˚C for 15 sec and at 60 ˚C for 60 sec. The gene expression levels of target genes: collagen II, Sox9 and aggrecan were determined based on the threshold PCR cycle-values (Ct(target)) following the instructions of Applied Bio-systems. The relative quantification was derived using the comparative CT method (also known as the 2^-ΔΔCt^ method), where the amount of target normalized to an endogenous control (beta tubulin) and relative to calibrator (samples without treatment). The specific primers designed for target genes are listed in Table 1.

**Statistical analysis. **All measurements were performed in triplicate. The data of MTT as well as PDT measurement were compared with one way ANOVA (SPSS version 19, IBM Corporation, Somers, NY, USA). Real time RT-PCR data was analyzed using Student t-test. A p value less than 0.05 was considered as statistically significant.

## Results

**Fig.1:**
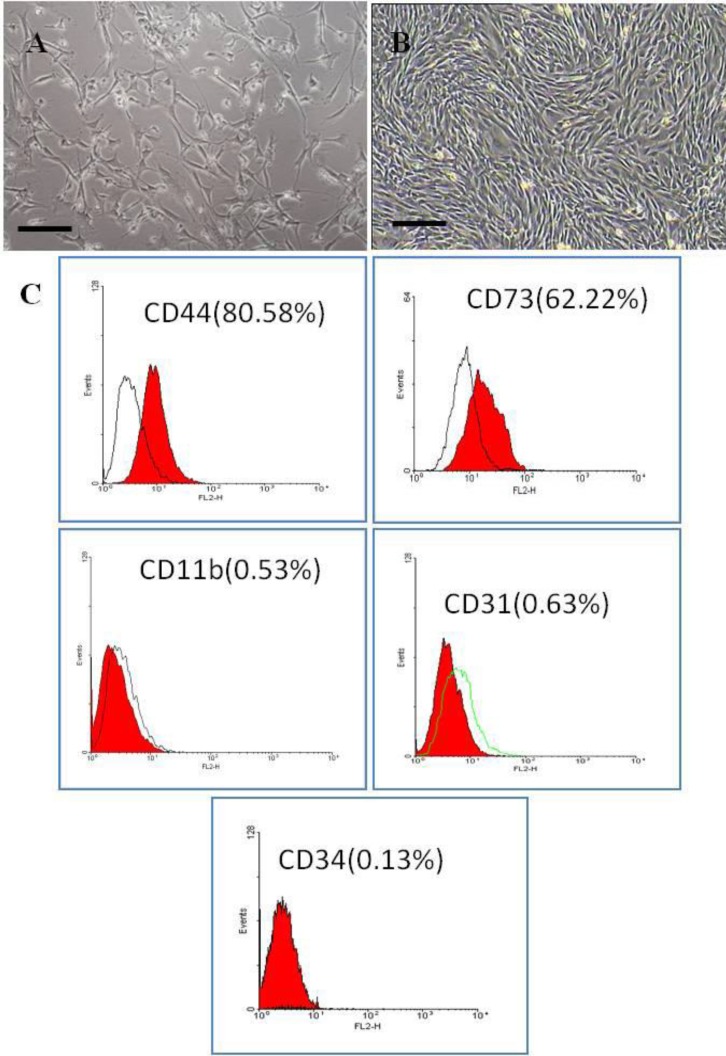
Mouse marrow-derived mesenchymal stem cells. **A)** The primary culture contained a variety of cell morphology (bar = 100 µm); **B)** Confluent culture was composed of fibroblastic cells (bar = 200 µm); **C) **Majority of the isolated cells expressed mesenchymal markers including CD44 and CD73. Hematopoietic (CD11b and CD34) as well as endothelial (CD31) markers were expressed on a very low percentage of the isolated cells.

**Cell culture. **The culture was daily observed with light inverted-contrast microscope. The primary culture tended to be heterogeneous containing elongated, triangular and flattened cells (Fig. 1A). Fibroblastic cells survived and dominated the culture. At day nine the culture became confluent having a monolayer of spindle-shaped fibroblast-like cells (Fig. 1B). Fibroblastic morphology was maintained throughout the cultivation period at passages. At sub-cultures the cells tended to rapidly proliferate reaching confluency in seven days.

**Flow cytometry. **According to our findings, the majority of the isolated cells tended to express mesenchymal markers including CD73 and CD44. Hematopoietic as well as endothelial cell markers such as CD31, CD11b and CD34 were expressed at very low percentage of the studied cells (Fig. 1C).

**Fig. 2:**
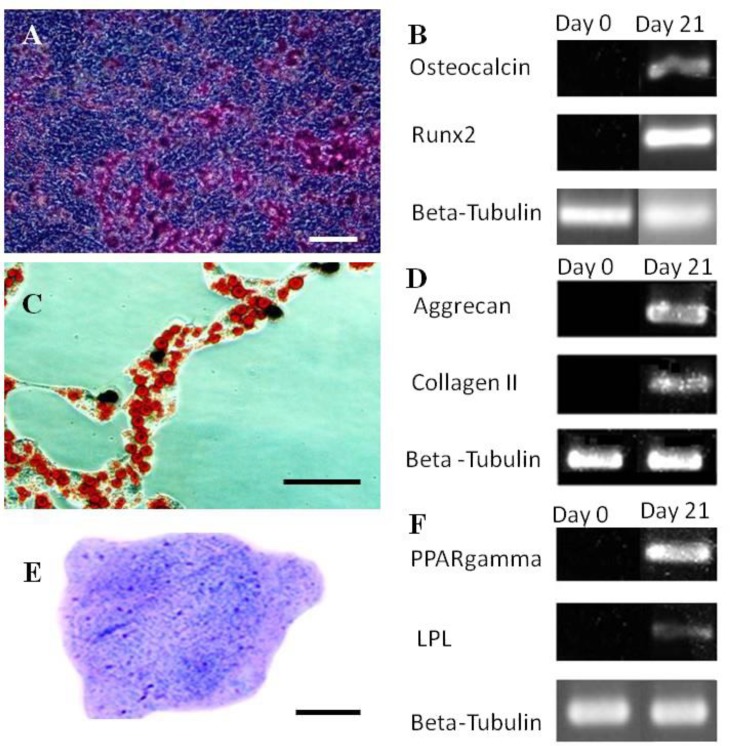
Multi-lineage differentiation of the isolated cells from mouse bone marrow. **A)** Osteogenic culture stained by alizarin red; **B)** Bone-specific genes expressed in the osteogenic culture; **C)** Adipogenic culture stained by Oil red; **D)** Adipose-related genes expressed in the adipogenic culture; **E)** Section prepared from chondrogenic pellets stained by toluidin blue; **F)** Cartilage-specific genes expressed in chondrogenic culture. Bar = 200 µm.

**Multilineage differentiation potential. **At osteogenic cultures the first morphologic changes were appeared a week after culture initiation as some cells aggregated into small nodules. The number of these osteogenic aggregates was then progressively increased. The osteogenic nodules tended to positively stain red with alizarin red staining (Fig. 2A). In addition RT-PCR analysis indicated that the bone specific mRNA including osteocalcin and Runx2 were produced at the cultures (Fig. 2B). At adipogenic cultures small lipid-like droplets were formed in some cells at day three. These cells gradually increased in number as the culture advanced in time. Following Oil red staining the droplets were stained red. Furthermore, according to the RT-PCR analysis the cells tended to express adipocyte-specific genes including peroxisome proliferator-activated receptor gamma (PPARgamma) and lipoprotein lipase (LPL). Sections prepared from the pellet of chondrogenic culture were successfully stained purple following tolouidin blue staining indicating that metachromatic matrix was produced in the culture. Also, based on the RT-PCR data chondrogenic pellets expressed cartilage-specific genes including collagen II and aggrecan.

**Fig. 3:**
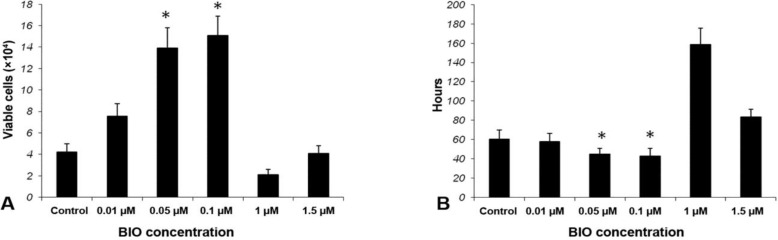
Cell viability and population doubling number (PDT) at cultures treated by BIO. **A) **The number of viable cells was significantly higher at culture treated by 0.1 and 0.05 µM BIO than the cultures with 0.01, 1.0 and 1.5 µM BIO and the control. * indicates a difference, (*p* < 0.5). There were no significant difference between the cultures with 0.1 and 0.05 BIO concentrations; **B)** The same relationships were observed among the studied cultures regarding PDN values

**Cell Viability. **According to MTT analysis, the number of viable cells in the cultures with 0.05 and 0.1 µM BIO were significantly more than that in the cultures without BIO (the control culture) as well as the culture with 0.01, 1.0 and 1.5 µM BIO (*p* < 0.05). Although the cell number was slightly higher in cultures with 0.1 than 0.05 µM BIO, the difference was not statistically significant (Fig. 3A).

**Cell proliferation : population doubling time. **Based on PDN calculations, at cultures treated with 0.1 µM BIO, the growth rate was significantly higher than the control culture and the cultures with 1.0, 1.5, 0.01 µM BIO (*p* < 0.05). While at cultures with 0.1 µM BIO, the cells tended to double their population in 43.07 ± 7.7 hr, the population doubling time for the cultures with 1.0, 1.5, 0.01 µM BIO and the control (without BIO) were 158.49 ± 17, 83.16 ± 8.4, 57.93 ± 8.6 and 60.21 ± 9.5 hr, respectively. The PDN for the culture treated with 0.05 µM BIO was 44.91 ± 6 which was one hour higher than that (PDN = 43.07 ± 7.7) for the culture with 0.1 µM BIO. The difference was not statistically significant (Fig. 3B).

**Fig. 4:**
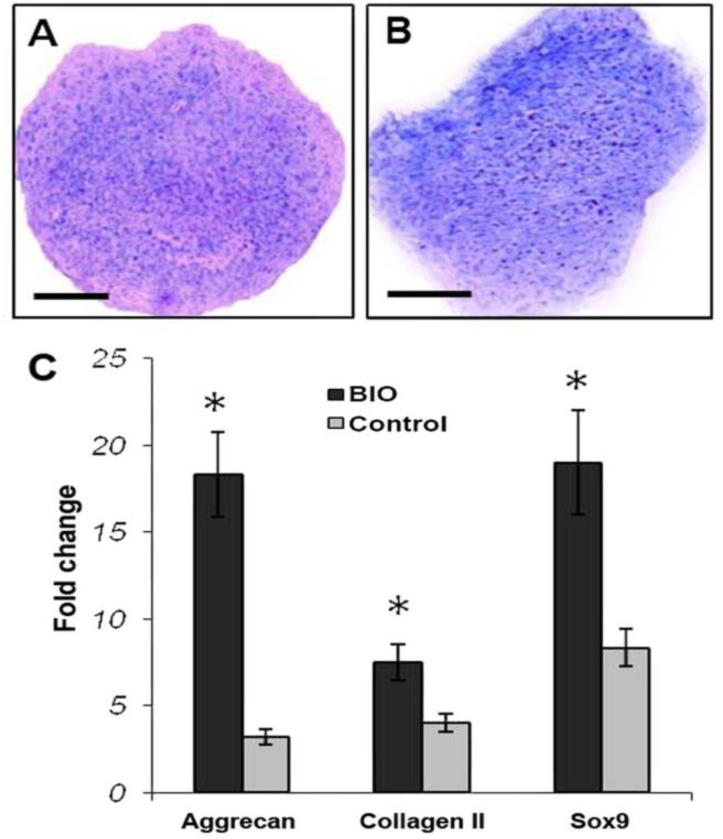
Chondrogenic culture of mouse marrow-derived MSCs treated by BIO. There was apparently more metachromasia (i.e. the intensity of purple stain) at cultures with BIO treatment **(A)** than without BIO **(B)**. This was in accordance with the real-time PCR findings indicating that cartilage-specific genes were significantly up-regulated at BIO-treated cultures, (Bar = 200 µm). **C)** Comparative analysis of fold change of cartilage-specific genes using quantitative PCR: The level of aggreacan, collagen and Sox9 expression in cultures with BIO was significantly higher than the culture without BIO (the control). * indicates a significant difference, (*p* < 0.05).

**Quantitative real-time RT-PCR. **According to this data (Fig. 4), at the cultures treated by BIO there was a significant up-regulation of the cartilage specific genes including aggrecan, collagen II and Sox9 (*p* < 0.05).

**Table 1:** Primers used in PCR

**Gene**	**Primer Sequence**	**Annealing Temperature (˚C)**
**RUNX2**	Forward: CAG CAT CCT ATC AGT TCC CAA Reverse: CAG CGT CAA CAC CAT CAT	60
**Osteocalcin**	Forward: GGC AAT AAG CTA GTG AAC AG Reverse: GGT CCT AAA TAG TGA TAC CGT	60
**LPL**	Forward :AAT TGT CCC ATG CTG TAA CC Reverse: CAG GAC ACA GGA AGC TAA GG	60
**PPAR-gamma**	Forward: GAG CAC TTC ACA AGA AAT TAC C Reverse: AAT GCT GGA GAA ATC AAC TG	59
**Col II**	Forward: GTT CAC ATA CAC TGC CCT Reverse: GTC CAC ACC AAA TTC CTC	60
**Aggrecan**	Forward: CCC AGA GAA ATT CAC CTT CC Reverse: TAGATA GAC AGTCCT TAC ACCC	60
**Βeta-Tubulin**	Forward: TCA CTG TGC CTG AAC TTA CC Reverse: GGA ACA TAG CCG TAA ACT GC	60

## Discussion

In this study, the effect of BIO on proliferation of mouse marrow derived MSC culture was investigated and the best concentration with mitotic effects was determined. According to our result the presence of 0.1 µM BIO in murine MSC culture medium resulted in the significant propagation of the cells. This data would be helpful for scientists involved at MSC-based tissue regeneration where enormous amount of stem cells would be needed to promote the reconstruction of lost tissues. Furthermore, we investigated whether a selected concentration with proliferative effects influence MSC chondrogenic differentiation. Interestingly, the presence of BIO in mouse MSC chondrogenic culture led to the significant enhancement of the cell differentiation. All these data highlights the crucial role of BIO in mouse MSC culture.

We have previously investigated the effect of BIO on proliferation of rat MSCs in culture. It seems that there is some differences among the rat versus mouse MSCs with respect to their response to BIO dosages. According to the present study, among the studied dosages of BIO, 0.1 µm tended to increase proliferation of mouse marrow MSCs in culture. This is in accordance with our former study on rat marrow MSCs.^21^ Furthermore, in this study we found an inhibiting effect of 1.0 µm BIO on murine MSC proliferation. This is not in accordance with that of our previous study indicating the proliferation-promoting effect of 1.0 µm BIO in rat MSC culture. This different response of MSCs to the same BIO concentration must have a logical explanation. One explanation would be that the behavior of MSC is species-specific. The different behavior of MSCs has been already reported at different species. For example it has been indicated that mouse MSCs are far more difficult both to isolate from bone marrow and to expand in culture than those of human or rat MSCs.^23^ Even MSCs isolated from bone marrow of different strains of inbred mice have been reported to vary in their surface epitopes, rates of proliferation, and differentiation potential.^24^ Interestingly, Ren *et al*. have indicated that the mechanisms of MSC-mediated immune-suppression vary among the MSCs from human and mouse.^25^ All these evidences are indicated that MSCs from different species would behave differently in culture and explain the different response of cultured rat and mouse MSCs to the same BIO concentration.

The selection and application of the range of BIO concentration, in this study, was performed with the consideration of similar previous studies. Sato *et al*. have reported that the presence of BIO at concentrations less than 1.0 µM enhanced murine embryonic stem cell proliferation in culture.^20^ Similarly, in a study by Sinha *et al*. it has been found that the presence of BIO at 0.01, 0.05, and 0.1 µM could increase proliferation of epithelial cells of murine kidney proximal tubule.^19^ Considering these reports, in this study, the certain concentrations of BIO including 0.01, 0.05, 0.1, 1.0 and 1.5 µM were selected and added to murine MSC culture in order to determine their possible enhancing/inhibiting effects on cell proliferation. Among applied BIO concentrations, we found that the 1.0 µM resulted in decreased cell number in mouse MSC culture.

This cytotoxic effect of BIO at 1.0 µM was not in accordance with the finding of Sato *et al*. who reported proliferative effects of BIO at that concentration.^20^ This discrepancy would be conceivable noticing difference of cell types that have been used in two studies (mouse MSC versus mouse embryonic stem cells). 

Enhancement of cell proliferation potential has long been desired by investigators since application of the cells in cell-based treatment of tissue defects requires considerable numbers of MSCs. In this context, various strategies have been employed. For example, it has been reported that treatment of MSC culture with growth factors such as fibroblast growth factor could result in their significant expansion.^26^ Growth factors are bioactive materials that are very costly. They must be regularly renewed at culture due to their short life time which makes culture process very expensive. In the present study, we treated MSC culture with BIO and found it a powerful proliferation-promoting additive to mouse MSC culture. The BIO that is derived from Trypan purple is less expensive than growth factors. BIO is a small molecule that is defined as low molecular weight organic compounds capable of binding with high affinity to a biopolymers such as proteins, nucleic acids, or polysaccharides altering their activity or function. The most important advantage of small molecule is that they can rapidly diffuse across cell membranes, reach intracellular sites of action and specifically target the signaling pathway.^27^

Studies have indicated that BIO effect on cell proliferation has been mediated through activation of Wnt signaling pathways.^19^^,^^20^ In the present study, we indicated treatment of murine MSC culture with BIO at 0.1 and 0.05 µM can enhance proliferation of MSCs. We did not investigate whether or not this effect was mediated with Wnt pathways which needs further investigations. Furthermore, our preliminary experiments demonstrated that BIO at 0.1 µM possessed chondrogenic effects. We did not check the effects of other concentrations of BIO on MSC chondrogenic culture. Moreover, in the present study, the chondrogenic effect of BIO was examined at the end of culture period. To expand our preliminary findings, further studies are needed to exactly determine different doses and also response times of BIO at MSC chondrogenic culture which is of great importance in case it comes to use the cells to promote regeneration in articular cartilage defects. 

In conclusion, taken together we indicated that BIO at 0.1 µM could enhance mouse MSC proliferation at culture. Furthermore, this concentration tended to enhance chondrogenic differentiation of MSCs. 
